# Mapping the anatomic distribution of digital artery perforators: a pilot cadaveric study for reconstructive flap surgery

**DOI:** 10.3389/fsurg.2025.1677597

**Published:** 2025-11-03

**Authors:** Rasyidah Rehir, Sameer Dhumale

**Affiliations:** 1 Anatomy, Edinburgh Medical School: Biomedical Sciences, University of Edinburgh, Old Medical School, Edinburgh, United Kingdom; 2 Department of Anatomy, Faculty of Medicine, Universiti Kebangsaan Malaysia, Kuala Lumpur, Malaysia

**Keywords:** anatomical pattern, flap surgery, digital artery, perforator, prevalence, reconstructive flap surgery, hand, microsurgery

## Abstract

**Introduction:**

Fingertip injuries, particularly crush injuries, are among the most common hand injuries across all age groups. The Digital Artery Perforator (DAP) flap has become a preferred option for fingertip reconstruction due to its simplicity, reliability, and minimally invasive nature compared to techniques like cross-finger or thenar flaps. Despite its growing use, detailed documentation of DAP patterns crucial for effective surgical planning is limited, especially in relation to demographic factors. This study aimed to map the anatomical distribution of DAPs and provide preliminary local data.

**Methods:**

Sixteen triphalangeal digits (excluding thumbs) from four female cadaveric upper limbs (mean age: 83 years) were injected with gelatin dye and dissected to expose DAPs. Photographs of the dissections were analyzed for DAP distribution across hand laterality, digit type, finger sides, phalanges, and phalanx thirds.

**Results:**

A total of 226 perforators were identified. On average, there were 57 DAPs per hand, 14 per digit, seven per finger side, five per phalanx, and two per phalanx third. Each phalanx contained at least one DAP, with an average of two DAPs per phalanx per finger side.

**Conclusion:**

High-density areas of DAP distribution suggest consistent anatomical patterns, supporting reliable DAP flap design. The findings indicate that preoperative Doppler ultrasound may not be necessary for flap planning, potentially simplifying surgical workflows. While limited by a small sample size, this study offers foundational insights for improving fingertip reconstruction and highlights the need for further research into DAP patterns and their clinical applications.

## Introduction

Fingertip injuries are common, accounting for about 10% of all accidents and two-thirds of hand injuries in children (1). These injuries can range from minor lacerations to amputations that cannot be reattached (2, 3). The most common type of fingertip injury is caused by crushing trauma (2–7). Fingertip injuries can lead to long-term complications, such as pain, abnormal sensation, deformities, and reduced strength, making timely and expert treatment essential (6–8).

Treatment of these injuries can include options like secondary intention healing, skin grafts, and local or free flaps (9). The ideal approach depends on factors such as surgeon experience, patient's characteristics, and the nature of the injury (10–13). The goal is to restore function, sensation, and appearance while avoiding donor site complications (1, 13, 14). For injuries where skin grafting isn't feasible, particularly if bones or tendons are exposed, local flaps are often used (6, 10, 15, 16).

One promising option is the Digital Artery Perforator (DAP) flap, which is based on the digital artery and is known for its reliability and suitability for fingertip reconstructions (15, 17–19). This flap has the advantage of being less invasive and technically simpler compared to other methods including cross-finger or thenar flaps, although it has limitations, including the difficulty in detecting perforators and the risk of flap failure due to its small size and short length (20–22). Apart from that, it is also indicated in patients with a more proximal defects particularly those resulting from Dupuytren's contracture, a progressive fibrosing disorder of the fingers and palm of the hand, or camptodactyly, a condition where one or more fingers are and cannot fully straighten (23, 24). Both conditions result in finger flexion, but they differ in their causes and locations.

Therefore, a better understanding of the DAP flap's perforator patterns could improve surgical outcomes by allowing more accurate preoperative planning. This could reduce surgery and recovery time, improve flap survival, and address some of the limitations associated with the DAP flap. A modification, the Predictable Pattern DAP (PPDAP) flap (25–30), eliminates the need for Doppler devices by mapping perforators' locations preoperatively using simple tools like a ruler and marker (31, 32). This innovation made selection of perforator for flap possible even without Doppler ultrasound.

We investigated the prevalence of DAP perforators and determined how the DAP pattern relates to surgical landmarks such as laterality, finger position, and phalanx.

## Materials and methods

This study used cadaveric specimens obtained from a local Scottish University, following ethical approval under the Human Tissue (Scotland) Act 2006 (Ethical code: ANATED_0035 and ANATED_0038). A total of 16 triphalangeal digits (excluding thumb) from four female cadaveric upper limbs (mean age 83 years) were examined. The digits included in this study had no visible trauma or signs of prior surgery.

Before perfusion, the specimens were thawed at room temperature for 24 h. The hand was massaged to drain blood and clots, then irrigated with warm saline through the radial and ulnar arteries using a catheter. After irrigation, the hand was massaged again to drain excess solution. To enhance the DAPs, a gelatine and methylene blue dye solution was perfused through the radial and ulnar arteries using 18G cannulas and 20cc syringes. The specimens were then sealed in biohazard bags and placed in a warm water bath for five minutes to allow the dye to spread. The dye solidified overnight at 5–7°C.

Dissection began on the palmar side of the hand, with midline longitudinal incisions made along the axis of each digit to expose the DAPs. The skin, subcutaneous tissue, tendons, ligaments, and joint capsules were dissected to reveal the neurovascular bundle and DAPs, which branched from the proper digital arteries. The perforators were counted manually, and localization was assisted by a surgical microscope. Digital callipers and a digital single-lens reflex camera (DSLR) camera were utilized for further photographic analysis. The distance of the DAP origin from the metacarpophalangeal (MCP), proximal interphalangeal (PIP), and distal interphalangeal (DIP) joints was measured in the image captured using Adobe Photoshop software, by comparing the distance in the image using a digital scale to the physical digital callipers to ensure standardized measurements. The same software was used to further determine the number of DAPs by dividing the phalanx into three equal parts (proximal, middle, and distal thirds) collectively referred to as the phalanx thirds. The number and location based on laterality, finger, finger side, phalanx, and phalanx third of the perforators were documented.

## Results

### Repeatability and validity test results (Kruskal-Wallis test)

This study demonstrated the repeatability and reliability of the measurement methodology for identifying DAPs in triphalangeal digits. The Kruskal–Wallis One Way Analysis of Variance on ranks was performed to test the repeatability and validity of measurements for Dorsal Artery Perforators (DAPs) in triphalangeal digits. The results showed no significant difference in measurements taken by a single observer at different times (*P* = 1.00), nor was there any difference between measurements made by different observers (*P* = 1.00). This indicated that the measurement methodology used in this study is both reliable and repeatable.

### Number of DAP in triphalangeal digits

**Figure 1:**
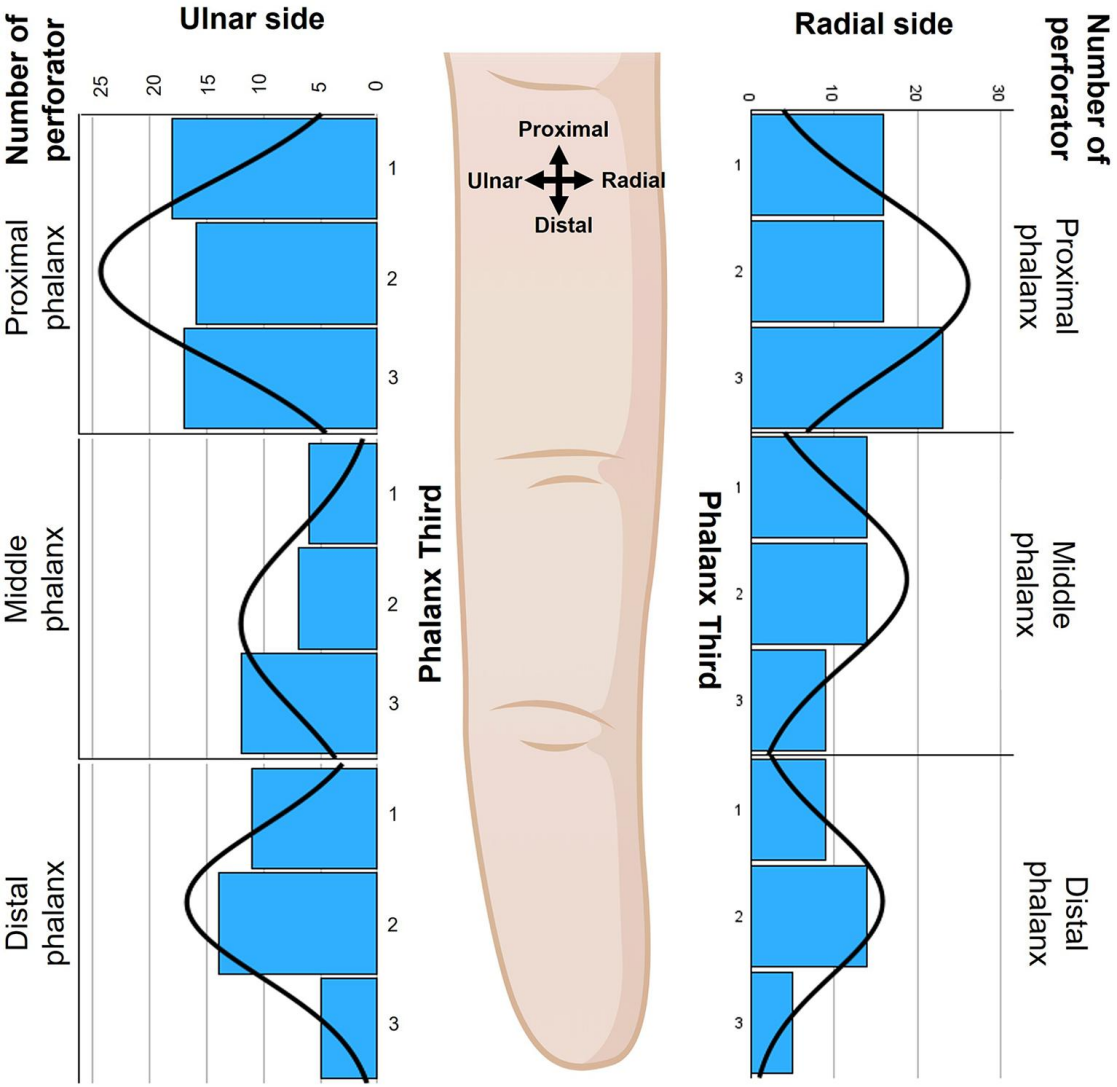
The distribution of DAP across each finger side, phalanx, and phalanx third (1: proximal third, 2: middle third, 3: distal third). The black curves in each graph represent the distribution of the DAPs across each phalanx and finger side. The image was created by author using Biorender.com.

A total of 226 DAPs were observed in 16 triphalangeal digits. The distribution of DAPs across various factors such as laterality, digits, finger sides, and phalanges was summarized in Table 1 and illustrated in Figure 1. In terms of hand laterality, 52% of DAPs were found in the left-hand specimens, and 48% were in right-hand specimens, with an average of 57 (Min. = 44; Max. = 64) DAPs per hand (Table 2). For digit distribution, 24% of the perforators were in digit two, 27% in digit three, 24% in digit four, and 26% in digit five, giving an average of 14 (Min. = 10; Max = 18) DAPs per digit. Radial sides had 53% of perforators, while ulnar sides had 47%, with an average of seven (Min. = 4; Max. = 11) DAPs per finger side. The DAPs were most commonly located in the proximal phalanx (47%), followed by the middle phalanx (27%) and distal phalanx (26%). Table 2 further shows that the DAP distribution on one finger side of each phalanx ranged from 15% to 17%, with an average of two (Min. = 2; Max. = 11) DAPs per phalanx and two (Min. = 1; Max. = 6) DAPs per phalanx on one side.

**Table 1:** Distribution of the number of DAP based on laterality, digits, finger sides, and phalanx.

Specimen no.		A				B				Total
Laterality		Right		Left		Right		Left		
Digit	Phalanx	Radial side	Ulnar side	Radial side	Ulnar side	Radial side	Ulnar side	Radial side	Ulnar side	
2	Proximal	2	2	6	4	5	6	2	3	**30**
	Middle	2	1	2	1	2	2	2	1	**13**
	Distal	2	1	2	2	1	1	1	1	**11**
3	Proximal	3	2	5	2	5	5	4	2	**28**
	Middle	3	1	3	2	1	2	4	1	**17**
	Distal	2	2	2	2	1	2	3	2	**16**
4	Proximal	2	2	2	2	4	4	3	2	**21**
	Middle	1	1	1	2	1	1	6	2	**15**
	Distal	3	2	1	2	1	3	2	3	**17**
5	Proximal	2	3	3	5	2	3	5	4	**27**
	Middle	1	1	2	2	4	4	2	1	**17**
	Distal	2	1	1	2	2	2	2	2	**14**
Total		**25**	**19**	**30**	**28**	**29**	**35**	**36**	**24**	**226**

Bold values represent the sum of perforators found in each phalanges across all digits or those found on either the radial or ulnar sides of the fingers in this study.

**Table 2:** DAP distribution based on laterality, digits, finger sides, phalanx, and phalanx on one finger side, including average DAP number per location.

Location	Description	Percentage of perforators present per location, %	Average number of perforators present per location
Hand laterality	Left	52	57 (Min. = 44; Max. = 64)
	Right	48	
Digit number	2	24	14 (Min. = 10; Max = 18)
	3	27	
	4	24	
	5	26	
Finger side	Radial	53	7 (Min. = 4; Max. = 11)
	Ulnar	47	
Phalanx	Proximal	47	5 (Min. = 2; Max. = 11)
	Middle	27	
	Distal	26	
Phalanx on one finger side	Proximal (radial)	17	2 (Min. = 1; Max. = 6)
	Middle (radial)	16	
	Distal (radial)	20	
	Proximal (ulnar)	16	
	Middle (ulnar)	16	
	Distal (ulnar)	15	

### Statistical analysis and group comparisons

Further analysis using independent t-tests and one-way ANOVA showed no significant differences in the number of DAPs between hand laterality (*P* = 0.51), digits (*P* = 0.59), finger sides (*P* = 0.10), and phalanx thirds (*P* = 0.39). However, there were significant differences found between the number of DAPs based on the type of phalanx (*P* < 0.01), indicating that phalanx type influences the number of perforators.

### Location of DAPs in triphalangeal digits

**Figure 2:**
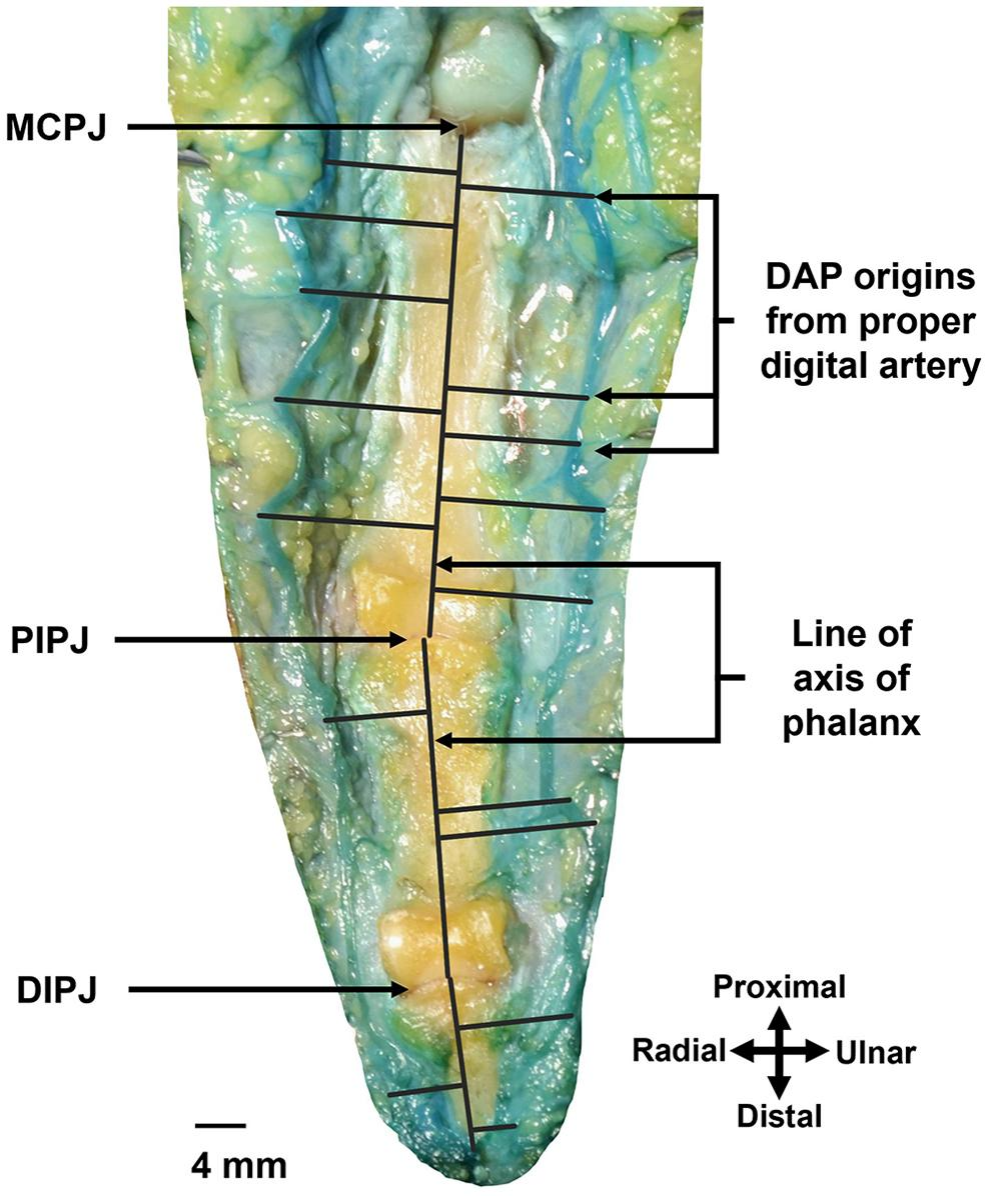
This is the right middle finger, as dissected from the palmar approach. Measurement of distance using Adobe Photoshop software of DAP origin from its proximal joints (MCPJ: MCP joint, PIPJ: PIP joint, DIPJ: DIP joint). Black lines drawn from DAP origin perpendicular to the vertical lines across the axis of the phalanges.

**Figure 3:**
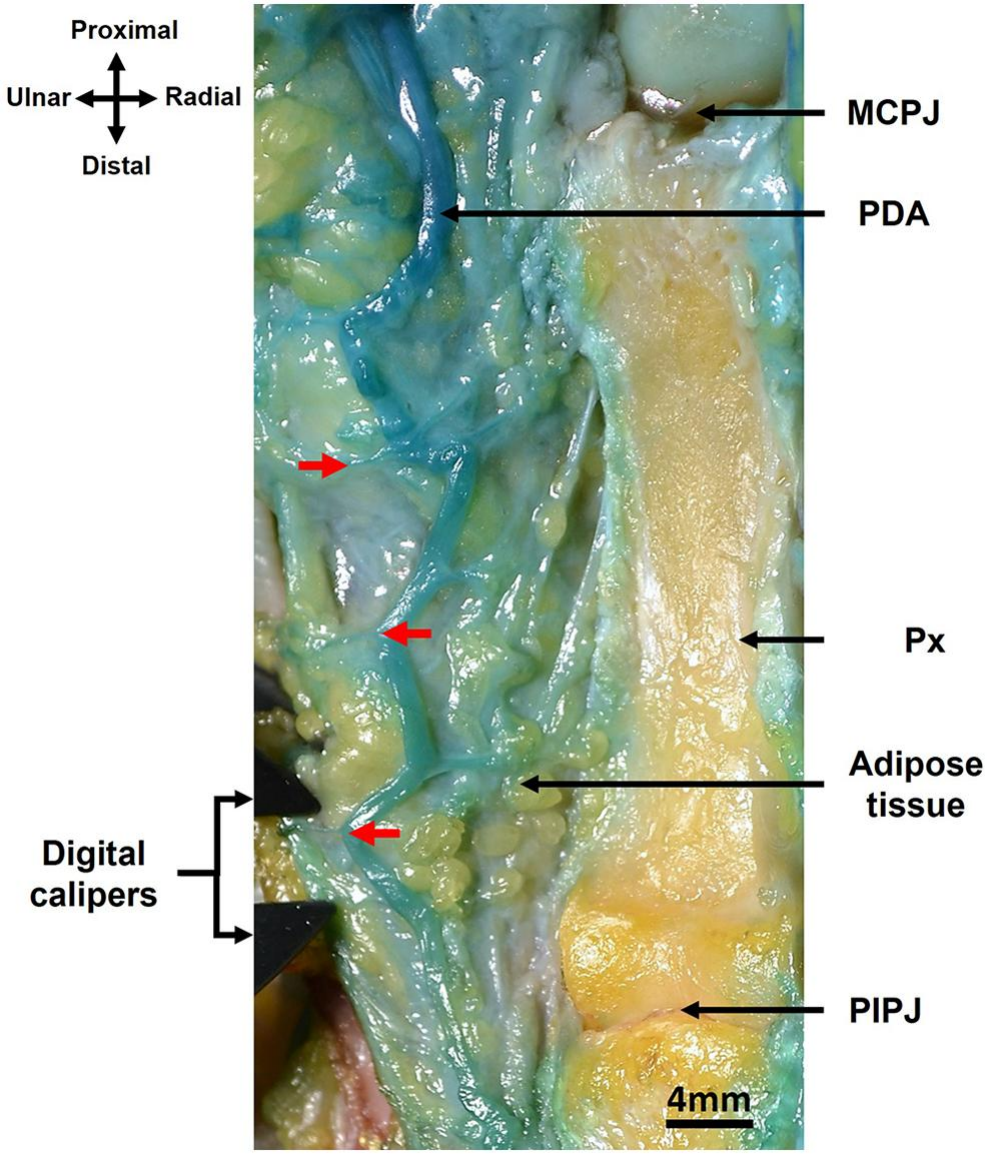
DAPs (red arrows) in relation to their origin, the proper digital artery (PDA) and MCP joint (MCPJ) in the proximal phalanx (Px) of right middle finger, as dissected from the palmar approach. The PIP joint (PIPJ) is also visible.

**Figure 4:**
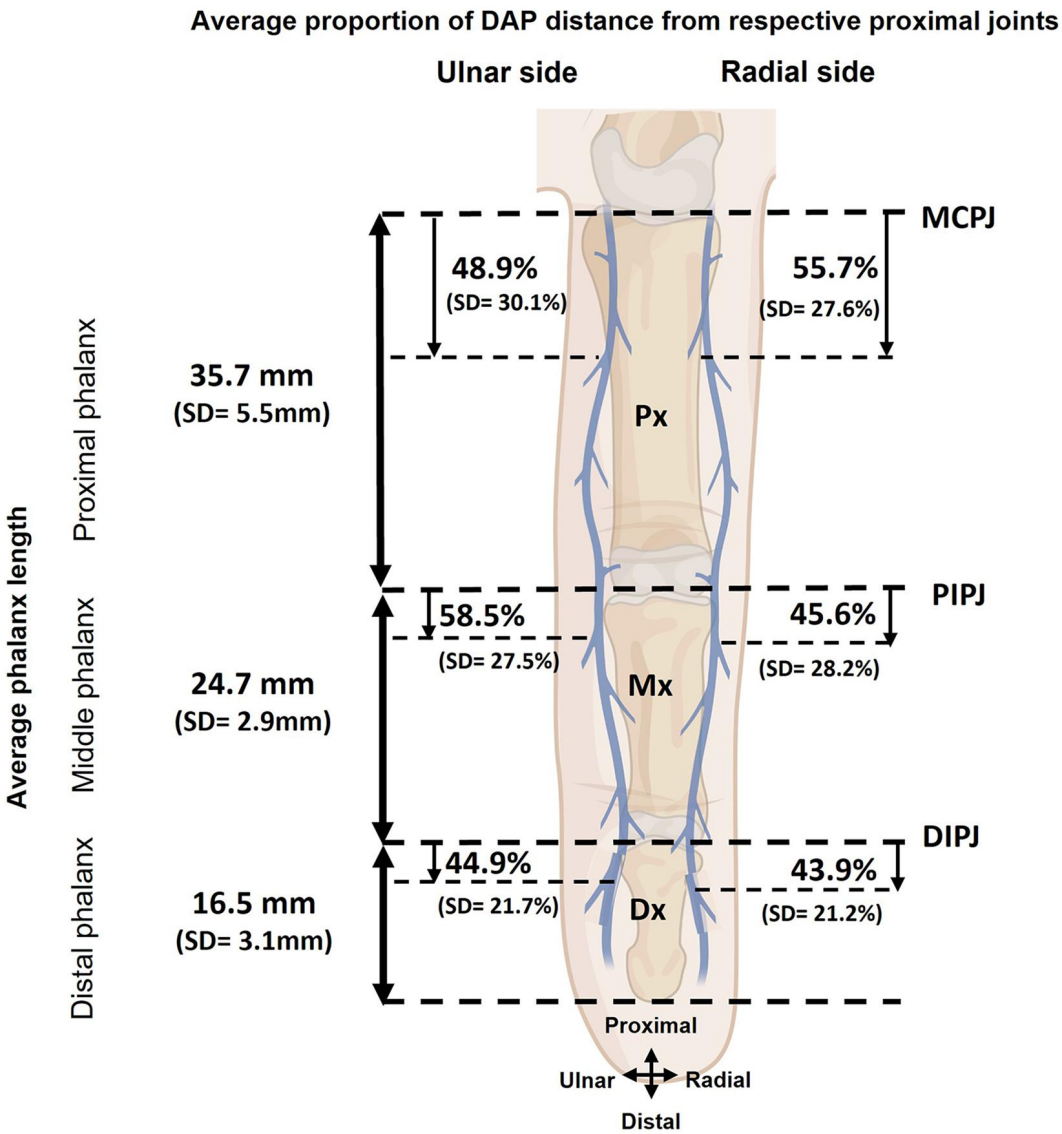
Average DAP distance from respective proximal joints (MCPJ: MCP joint, PIPJ: PIP joint, DIPJ: DIP joint) based on radial and ulnar sides. The image was created by author using Biorender.com. SD, standard deviation.

The location of DAPs in proximal, middle and distal phalanges were described as distance of DAPs originating from the proper digital artery to their respective proximal joints (MCP, PIP, and DIP joints) (Figures 2, 3). The mean or average distances were summarized based on hand laterality, digits, finger sides, and phalanx in Table 3 and were illustrated in Figure 4. Essentially, the mean distances of DAPs were furthermost in proximal phalanx from MCP joint, 18.8 mm (SD = 10.9 mm), followed by 12.4 mm (SD = 6.9 mm) in middle phalanx from PIP joint, and least in distal phalanx from DIP joint, 7.2 mm (SD = 3.5 mm). Figure 4 showed the mean DAP distances in proximal, middle and distal phalanges from respective proximal joints (MCP, PIP, and DIP joints) were 17.1 mm (SD = 10.9), 14.3 mm (SD = 6.6 mm), and 7.3 mm (SD = 3.4 mm) on radial side whereas, on ulnar side, the mean distances were 20.4 mm (SD = 10.8 mm), 11.2 mm (SD = 6.8 mm), and 7.1 mm (SD = 3.6 mm) respectively, showing similar pattern of distance, from longest to shortest.

**Table 3:** Average DAP distance from its proximal (MCP, PIP, and DIP) joints based on laterality, digits, phalanx on one finger sides, and phalanx.

Location	Description	Mean of DAP distance from respective proximal joints (SD), mm		
		MCP	PIP	DIP
Hand laterality	Right	18.4 (10.7)	13.0 (7.3)	7.3 (3.3)
	Left	19.3 (11.3)	12.0 (6.4)	7.2 (3.6)
Digit	2	19.0 (9.7)	10.9 (6.4)	6.2 (2.7)
	3	22.5 (12.2)	11.7 (6.1)	7.1 (3.6)
	4	19.7 (11.9)	14.3 (8.1)	9.0 (3.8)
	5	14.1 (8.8)	12.7 (6.9)	6.0 (2.9)
Finger side	Radial	20.4 (10.8)	11.2 (6.8)	7.1 (3.6)
	Ulnar	17.1 (10.9)	14.3 (6.6)	7.3 (3.4)
Phalanx	Proximal	18.8 (10.9)	NA	NA
	Middle	NA	12.4 (6.9)	NA
	Distal	NA	NA	7.2 (3.5)

SD, standard deviation; NA, not available.

### Comparison of DAP distance based on various factors

Further comparisons of the mean distance of DAP origin from the proximal joint revealed no significant differences between hand laterality (*P* = 0.97), digits (*P* = 0.18), and finger sides (*P* = 0.57). However, there were significant differences in the mean distance based on phalanx (*P* < 0.01) implying that the DAPs in proximal phalanx located further from the MCP joint than those in the middle and distal phalanges.

## Discussion

The DAP flap procedure is widely used in reconstructive surgery, but identifying the perforator arteries remains challenging. A better understanding of DAP perforator patterns could improve surgical outcomes by enabling more accurate preoperative planning. This study focused specifically on factors like the number, distribution and location of perforators.

There were several limitations in this study. First, it was based on a small sample of female cadavers. Since men are more likely to suffer fingertip injuries, especially in children and adults (3, 7), further studies should include male cadavers to explore potential sex differences. The average age of the cadavers was 83, meaning the findings may not be fully applicable to younger, working-age adults or children, who are more prone to fingertip injuries (3, 7). Apart from that, undiagnosed vasculopathy could be a limitation for the subjects of this age group (33, 34) and may alter findings of this study. Future studies should include larger sample size, male gender, consider a wider range of age groups, and data on comorbidities that may contribute to varied vascular pattern.

Additionally, the small sample size limited the ability to study more variables, such as the diameter of the vessels and branching patterns, which are critical for DAP flap survival.

The results of this study were consistent with previous research on DAP perforator patterns, such as a study by Navio-Fernandez et al. (35). Both studies found consistent anatomical patterns across sex, laterality, finger position, and phalanx type. Navio-Fernandez et al. reported an average of 7 DAPs per finger side, which is similar to the average DAP count per finger in this study (35).

In terms of methodology, the studies differed in their vascular visualization techniques. Navio-Fernandez et al. (35) used red latex injections, while this study used a gelatine and methylene blue mixture. Both studies cannulated the radial and ulnar arteries using similar techniques, but this study approached the DAPs from the palmar side, whereas the other study used a dorsal approach. These differences may explain some of the variations in the results. The gelatine-based dye used in this study might be more effective than latex in enhancing the DAPs. Although the Spanish study (35) had a larger sample size (112 digits from both sex), this study's smaller sample (16 digits from female cadavers) still detected a higher number of DAPs. Both studies found that most DAPs were located in the proximal phalanx and concentrated in the distal third of the phalanx. This pattern is consistent across different fingers and positions, reinforcing the predictability of DAP perforator distribution.

The distribution of DAPs was relatively consistent across laterality, digits, finger sides, phalanx, and phalanx thirds. While limited by a small sample size, these findings can provide valuable insights for surgical planning, particularly in reconstructive procedures that use DAP flaps however, further investigation is needed to determine whether preoperatively planning just from perforator mapping improves clinical outcome and a bigger number of samples is warranted to confirm this DAP distribution for the Scottish population.

The evolution of perforator flaps, including the DAP flap, has been significant since its introduction in the late 1980s (36, 37). The development of Doppler ultrasound technology improved the identification of perforators, and the concept of free-style perforator flaps emerged. The DAP flap was described in 2006, initially relying on Doppler ultrasound for preoperative localization (20, 38). Although inexpensive, widely available and easy to use, Doppler detection is often less reliable in detecting small-calibre vessels like DAPs (25–30). While Doppler devices are useful, they have limitations in detecting DAPs, as shown in studies like Shintani et al. (39), which found that Doppler ultrasound detected only three to four DAPs per digital artery, compared to 14 DAPs per finger side found in cadaveric studies.

However, studies like this does not eliminate the need for Doppler but, emphasize on the usefulness of mapping the DAP perforators based on anatomical landmarks, improving the predictability of flap harvesting. The findings of this study support the idea that DAP perforators follow consistent anatomical patterns. Mapping DAPs based on their anatomical locations is possible in cases where Doppler ultrasound is not available. Future studies can look into the reliability of perforator mapping and its clinical outcome in DAP flap reconstruction. Comparison of DAP flap outcome between perforator mapping and the use of Doppler ultrasound in preoperative planning can also be further investigated.

## Data Availability

The original contributions presented in the study are included in the article/Supplementary Material, further inquiries can be directed to the corresponding author.
